# An intelligent approach for Arabic handwritten letter recognition using convolutional neural network

**DOI:** 10.7717/peerj-cs.995

**Published:** 2022-05-27

**Authors:** Zahid Ullah, Mona Jamjoom

**Affiliations:** 1 Information Systems Department, Faculty of Computing and Information Technology, King Abdulaziz University, Jeddah, Saudi Arabia; 2 Department of Computer Sciences, College of Computer and Information Sciences, Princess Nourah Bint Abdulrahman University, Riyadh, Saudi Arabia

**Keywords:** Arabic handwritten letter, Recognition, Intelligent model, CNN, Evaluation

## Abstract

Currently, digital transformation has occurred in most countries in the world to varying degrees, but digitizing business processes are complex in terms of understanding the various aspects of manual documentation. The use of digital devices and intelligent systems is vital in the digital transformation of manual documentation from hardcopy to digital formats. The transformation of handwritten documents into electronic files is one of the principal aspects of digitization and represents a common need shared by today’s businesses. Generally, handwriting recognition poses a complex digitization challenge, and Arabic handwriting recognition, specifically, proves inordinately challenging due to the nature of Arabic scripts and the excessive diversity in human handwriting. This study presents an intelligent approach for recognizing handwritten Arabic letters. In this approach, a convolution neural network (CNN) model is proposed to recognize handwritten Arabic letters. The model is regularized using batch normalization and dropout operations. Moreover, the model was tested with and without dropout, resulting in a significant difference in the performance. Hence, the model overfitting has been prevented using dropout regularization. The proposed model was applied to the prominent, publicly-available Arabic handwritten characters (AHCD) dataset with 16,800 letters, and the performance was measured using several evaluation measures. The experimental results show the best fit of the proposed model in terms of higher accuracy results that reached 96.78%; additionally, other evaluation measures compared to popular domain-relevant approaches in the literature.

## Introduction

The world today is embracing digitizing documents through the adoption of various automated handwritten document recognition techniques (Balaha et al., 2021). In addition, an automated handwritten recognition system takes part in many ancillary applications, such as: educational applications (Altwaijry & Al-Turaiki, 2021), friendly learning environment (Al-Helali & Mahmoud, 2017), bank cheque handling, reading application forms, postal address handling, and handwriting-to-speech transformation (Ahmed et al., 2020). Consequently, handwriting recognition remains an active research area in recent decades and lots of handwriting recognition systems have been introduced to recognize different languages, of which the most common were English (Yuan et al., 2012), Chinese (Xiao et al., 2017; Zhong, Jin & Feng, 2015; Bai et al., 2014), French (Xiao et al., 2020), Urdu (Ali et al., 2020), and Arabic (Younis, 2017; Shams, Elsonbaty & El Sawy, 2020; Balaha et al., 2021). Furthermore, different research studies have proposed various intelligent techniques for handwriting recognition using machine learning methods, wherein some research has focused on digits, characters, text, or these elements in combination (Mudhsh & Almodfer, 2017). Moreover, some studies have applied their systems offline, such as in Ahmed et al. (2021), Yuan et al. (2012) and Abdalkafor & AlHamouz (2016) while others apply them online (Al-Taani & Al-Haj, 2010; Al-Helali & Mahmoud, 2017).

Currently, Arabic is one of the most common international languages used in the Middle East and North Africa (Javed, 2013), and one of the most widespread spoken languages in the world; its speakers exceed half a billion over the world (Altwaijry & Al-Turaiki, 2021). Moreover, many languages use Arabic letters, words, and sentence structures, such as Urdu, Pashto, Persian, and Jawi (Younis, 2017; Balaha, Ali & Badawy, 2021). The standard written Arabic language consists of 28 letters which are usually written starting from right to left and were derived from the script of the holy Quran (Javed, 2013; Balaha, Ali & Badawy, 2021). Some letters may look different if they occur at the start, middle, or end of the word; that is, the shape of the letter is changed based on its location in the word (Al-Taani & Al-Haj, 2010).

A general problem facing any handwriting recognition language system is the variability of the font and the letter dimension across the handwriting from one individual to another (Sahloul & Suen, 2014). Some studies have reported during data collection that numerous variabilities can be observed in the handwriting of the same individual (Almansari & Hashim, 2019), thus automatic recognition of Arabic letters remains a challenge (Mustapha et al., 2022). Likewise, Arabic handwriting recognition poses many challenges because of: (1) high diversity in human handwriting, (2) shortage of large and public datasets, (3) the nature of Arabic script (*e.g.*, cursive form, variable letter size, different letter’s shape depends on its location…etc.) (El-Sawy, Loey & EL-Bakry, 2017; Al-Taani & Al-Haj, 2010), (4) diverse styles in handwritten (Al-Helali & Mahmoud, 2017) and (5) There is a wealth of handwritten Arabic documents available in libraries (Balaha et al., 2021).

This study presents an intelligent approach to handwritten Arabic letter recognition using CNN. The proposed model developed in this approach was applied to a publicly available prominent dataset, referred to as AHCD. The proposed model has to recognize the 28 Arabic letters that exist in the dataset. The experimental results show the high performance of the proposed model applied to the AHCD dataset.

The remainder of this paper is ordered as follows: the next section discusses the literature review followed by step-by-step methodology, the next is the experimental setup followed by results and discussion, and the last section concludes this study.

## Literature Review

In this section, we summarized the works in the literature which introduced different methods for Arabic handwritten letter recognition. We focused on single letter recognition since it’s our concern in this study. Sahloul & Suen (2014) believed that achieving good accuracy was mainly based on the features selection during the preprocessing phase in the handwriting character recognition process. For that, they have used the CENPARMI database with 11,620 letters and extracted statistical, morphological and topological features. The method has shown a recognition rate of about 88% for all letters. In Abdalkafor & AlHamouz (2016), used the same dataset with a novel feature selection approach and MLP neural network and reported the model accuracy of 94.75%.

Similarly, Elleuch, Maalej & Kherallah (2016) have proposed an integrated model of two classifiers: CNN and Support vector machine (SVM) and used dropout for securing the model from overfitting. They found that CNN-based SVM with dropout is more efficient in recognizing the Arabic letters than that without dropout. Furthermore, they have tested the model on Handwritten Arabic characters (HACDB) and IFN/ENIT datasets with dropout operation and found error classification rates of 5.83% and 7.05% respectively.

In El-Sawy, Loey & EL-Bakry (2017) the authors have developed a CNN model for Arabic letter recognition. The authors have utilized multiple optimization methods for enhancing the performance of the model. The model was employed on the AHCD dataset and reported a testing accuracy of 94.9%. Younis (2017) introduced a deep neural network model using a robust CNN for handwritten Arabic character recognition. The proposed CNN used the regularization parameter for overfitting avoidance. The proposed model was applied to AIA9K and AHCD datasets and reported the classification error for both datasets of 5.2% and 2.4%, respectively.

Boufenar, Kerboua & Batouche (2018) investigated the applicability of the proposed DCNN for the recognition of offline handwritten Arabic letters using a transfer learning strategy. They have tested the proposed model on OIHACDB and AHCD datasets. The authors of this study have enriched the AHCD datasets with additional samples in the training set and reported a high level of efficacy of the proposed method. Elleuch & Kherallah (2018) has presented a deep learning based on SVM (DeepSVM) model for recognizing handwritten Arabic letters. In this regard, multi-label SVM and RBF kernel were used for testing Arabic letters in the HACDB dataset. The authors concluded with the effectiveness of the proposed method in terms of the experimental results and reported an error classification rate of 8.64%. Almansari & Hashim (2019) have presented a deep learning architecture with CNN and MLP neural network for recognizing isolated handwritten Arabic letters. The architecture performance was evaluated using the AHCD dataset and concluded with a testing accuracy of 95.27% of the architecture.

Another attempt was made by Ali & Suresha, (2019) for recognizing the Arabic handwritten letters using machine learning methods. An integrated recognition system based on CNN, k-Nearest Neighbour (kNN), and SVM was developed, and the accuracies analysis and classifiers’ performance were measured. The integrated recognition system was applied to AHDB and IFN/ENIT databases and reported an accuracy of 98.4%. This is worth noting that the datasets used were different from our study.

In Shams, Elsonbaty & El Sawy (2020), the authors proposed a method to recognize, detect, and validate input handwritten Arabic characters using deep CNN and SVM. The proposed method attempted to check the similarity between the input characters and the stored characters. It used a clustering method specifically k-means for recognizing multi-stroke characters and applied the proposed method on 840 tested images. The reported result of model accuracy was 95.07%. Djaghbellou et al. (2020) has proposed a new framework based on multi-scale histogram oriented gradient (HOG) features and the deep rule-based classifier (DRB) for recognizing handwritten Arabic letters. The framework integrates multi-scale HOG during feature extraction and then DRB is used to classify the comprehensive HOG features and predict the class labels. The framework was applied to the AHCD dataset and reported the recognition rate of 74.61% which was comparatively higher than other studies.

A larger dataset named “HMBD” that contains 54,115 characters has been used by Balaha et al. (2021). After multiple attempts, they selected the best 14 native CNN architectures that differ in a hierarchy of constructing the architecture layers, the best of them resulted in a testing accuracy of 91.96%. Furthermore, more improvement has been done using optimization methods (*e.g.*, transfer learning (TF) and genetic algorithm (GA)) that led to achieving a testing accuracy of 92.88%. Ali & Mallaiah (2021) have proposed a model of CNN based-SVM with dropout for Arabic handwritten letter recognition and tested on several datasets such as AHDB, AHCD, HACDB, and IFN/ENIT. The authors reported promising results of the proposed model in comparison to other models built for the same domain. The model achieved an accuracy of 96.80% applied on AHDB.

**Figure 1:**
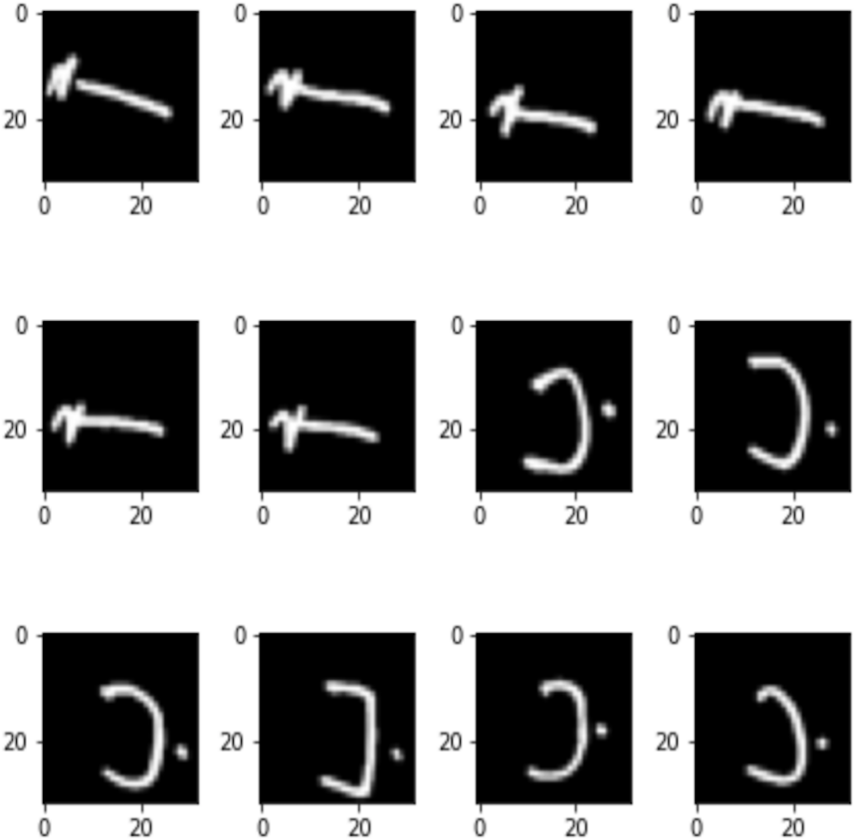
Sample of Arabic letters in a training set.

**Figure 2:**
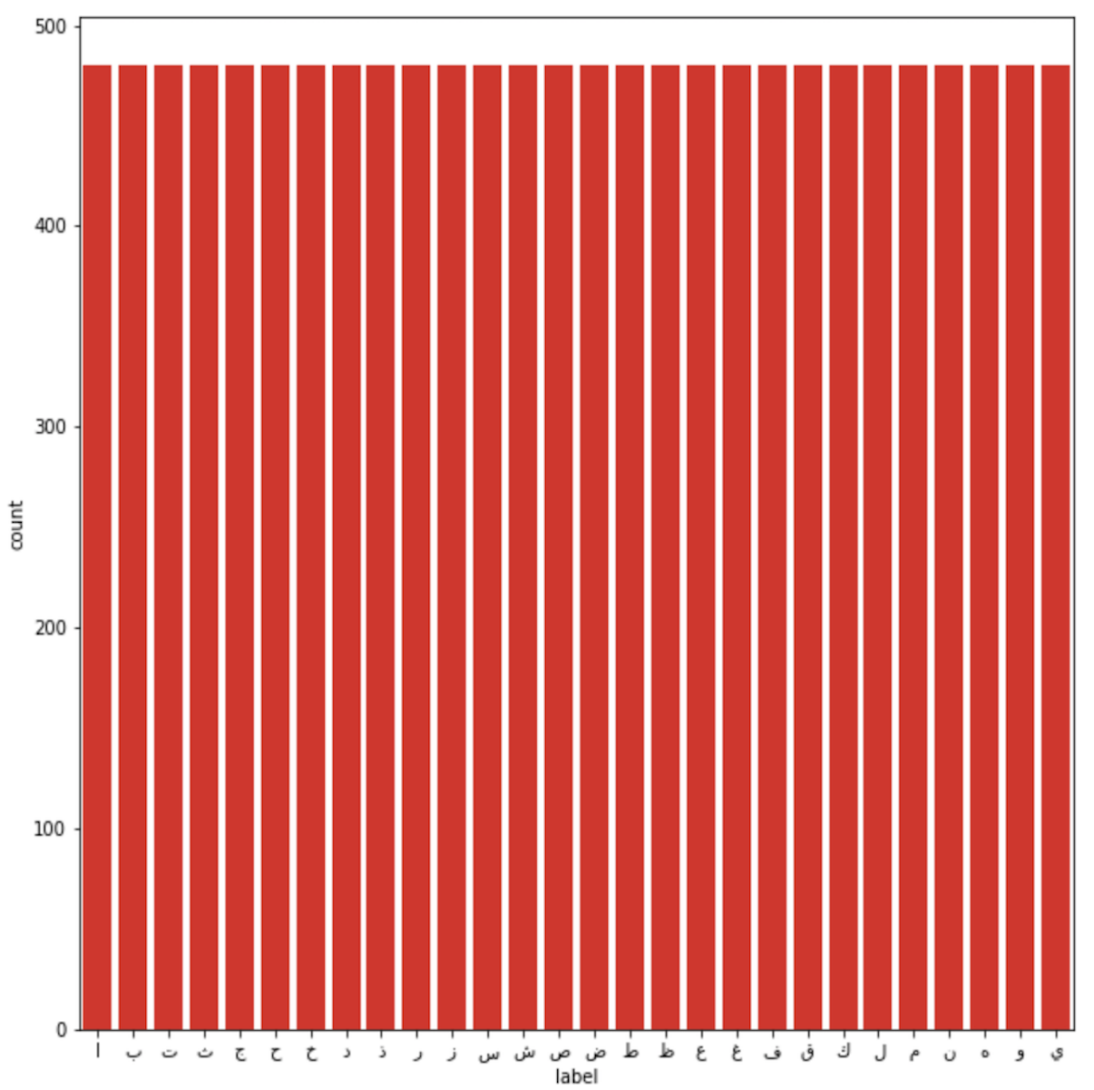
Training images per class.

**Figure 3:**
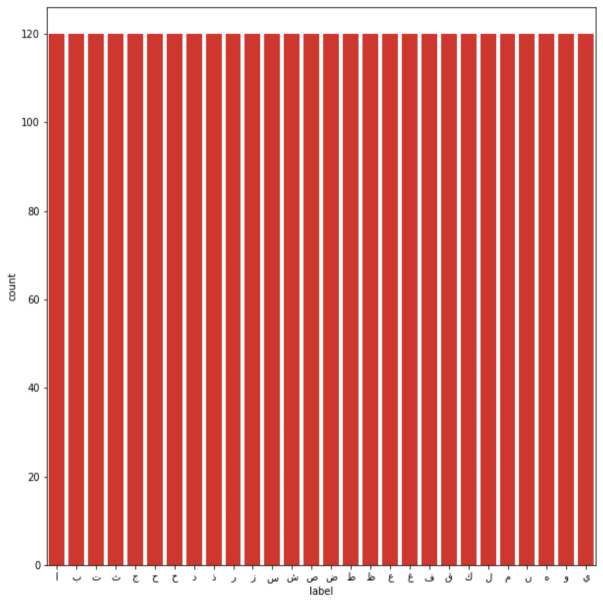
Testing images per class.

A novel supervised CNN model was introduced by Ahmed et al. (2021) that extracts optimal features and applied batch normalization and dropout regularization layers for enhancing the overall performance. The model has shown excellent classification results on a diverse set of six benchmark databases, including MADBase (Digits), CMATERDB (Digits), HACDB (Characters), SUST-ALT (Digits), SUST-ALT (Characters), and SUST-ALT (Names). Although, the accuracy of this model is above 99% but the model was not tested on the AHCD dataset.

An attempt was made by Aljarrah, Zyout & Duwairi (2021) who proposed a CNN model for recognizing the Arabic offline letters. The proposed model was trained using the AHCD dataset and achieved high performance model with an error classification rate of 2.3% after applying data augmentation. Subsequently, the authors (Altwaijry & Al-Turaiki, 2021) evaluated their offline model for recognizing Arabic letters based on CNN architecture using two datasets: Hijja which contains 47,434 images of single Arabic characters written by children aged 7–12 years and AHCD containing 16,800 images. The performance of the model shows an error classification rate of 3% and 12% for AHCD and Hijja, respectively.

## Materials & Methods

### Data collection

The dataset used to conduct this study was the Arabic handwritten characters dataset (AHCD) harvested from Kaggle (El-Sawy, Loey & EL-Bakry, 2017). The dataset consisted of a total of 16,800 handwritten letters collected from 60 people ranging in age from 19 to 40 years. The dataset comprised 4 separate files (*i.e.,* train images, training labels, test images, and testing labels). The authors have further explained that 90% of the people were right-handed. The total number of Arabic class labels used in AHCD is 28 (*i.e.,* from ‘alef’ to ‘yaa’). During the data collection, each person was required to provide a set of 28 letters repeatedly 10 times. Figure 1 shows sample letters in the AHCD dataset. Moreover, the 16,800 letters were split into two sets in that 80% of the letters were in training and the rest of 20% were in the test set. The training set consisted of 13,440 letters divided into 480 images per class and the test set of 3,360 letters divided into 120 images per class as shown in Figs. 2 and 3 respectively.

### Dataset preprocessing

Data preprocessing is an essential step in enhancing the quality of data by removing “garbage” (Al-Mudimigh & Ullah, 2011) and preparing it for best-fitted model development (Ullah & Al-Mudimigh, 2012). In this study, firstly, the images were reshaped in size to 32 × 32. According to Ghosh, Das & Nasipuri (2019), CNN is normally accepting fixed size images that leads to several challenges in data acquisition and model development; therefore, the input image reshape is performed to overcome such challenges and fed to the network. Similarly, flipping and rotations were performed for better performance and then reshaped the whole array to a size of 32 × 32 × 1. The 32 ×32 × 1 size image represents a greyscale image. Moreover, the images were also normalized (or simply divided by 255). The categorical class labels were also executed in that integer values that represent different categories were transformed into a matrix of binary values (Ullah & Jamjoom, 2022). For a better understanding of the classifier, the index of the class labels should start at 0 rather than 1 in the original dataset; therefore, the start of the class label is set to 0, thus the label ranges from 0 to 27 for all 28 classes. Finally, the training and testing sets were reshuffled for increasing the likelihood of the best fitted model.

### Convolutional neural network (CNN)

CNN is one of the most commonly used kinds of artificial neural networks (ANN) (Acharya et al., 2017) that has shown extraordinary contribution in image related problems (Altwaijry & Al-Turaiki, 2021). CNN is typically consisted of three major layers such as convolution, pooling and fully connected layers (Sultana, Sufian & Dutta, 2018) and an output layer.

In the convolution layer of a CNN, an image is convolved with filters to create feature maps (Yu, Jia & Xu, 2017) which forward these maps to the subsequent layer for extracting another higher-level of feature set from the input image (Abiwinanda et al., 2019). In this layer, most of the filters work concurrently on the same input image while each filter identifies a distinct parameter (Singh & Shankar, 2021). Furthermore, the common activation function used in this layer addressed non-linearity such as sigmoid, tanh, ReLU, and leaky ReLU (Yu, Jia & Xu, 2017).

A pooling layer is normally used between the convolution layers for reducing the dimensionality of an image while keeping the foremost features in the feature maps (Abiwinanda et al., 2019). The dimensionality reduction happens in two ways, either by selecting the maximum region or average region of an image (Singh & Shankar, 2021). Moreover, pooling reduces the image size but keeps the predominant features (Tekerek, 2021). A flattening layer is used to vectorize the feature maps which is normally occurs right after the last convolution layer. The flatten input vector passed into the fully connected layers which output the predicted class based on the correctly classified samples at each output neuron (Abiwinanda et al., 2019). Moreover, the activation function used on the fully connected layer is ReLu, sigmoid, etc., except for the output layer which is softmax that gives the output of the predicted class.

In this study, a hyperparameter tunning was performed using grid parameter tuning. This method suggests all possible combinations of hyperparameters. The optimal combination of the hyperparameters is then selected using the selected best fitted model.

### Proposed CNN Model

**Figure 4:**
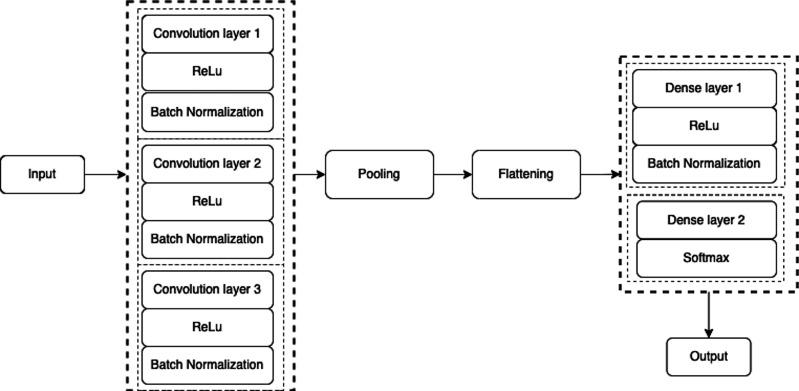
A schematic architecture of CNN.

**Figure 5:**
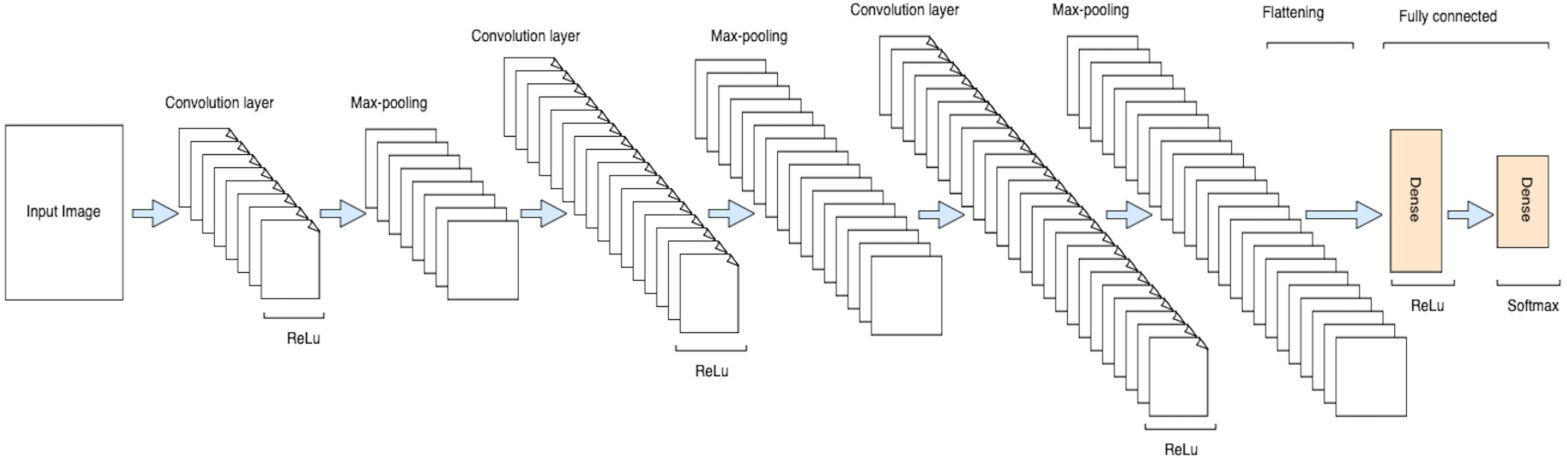
Architecture of the proposed CNN model.

The proposed CNN model is consisted of a total of eight layers including three convolution layers, three max-pooling layers, and two fully connected layers as shown in Figs. 4 and 5. The input layer takes the input in three dimensions that are height, width, and dimension (h × w × d). The *D* = 3 represents the RGB image while the *D* = 1 shows grey scale image. In the proposed model, the input image was a grey scale image of size 32 ×32 × 1. In each convolution layer, the layers are convolved with respect to the kernel size of (3 ×3). The small kernel size of (3 ×3) is appropriate for this classification process. In each convolution layer, the activation function used was ReLu for non-linearity purposes and padding was used to prevent the image shrinkage.

(1) \begin{eqnarray*}ReLu~ \left( z \right) =\max \nolimits ~(0,z).\end{eqnarray*}

In CNN, the input layer provides only the image shape without any learning; therefore, the number of parameters is 0. The actual learning of the CNN model starts at the convolution layers. In this model, the input image is provided in a grey scale of shape 32 ×32 × 1. The first convolution layer used 32 features, kernel size of (3 ×3), strides (*s* = 1), and same-padding of size 1. The output shape of the layers is calculated as (filters + 2 × padding –(kernel_size - 1)). Thus, the output shape of the first layer is 32 × 32 × 32. The activation size is the dot product of the output shape, resulting in the first layer activation size of 32,768 elements. Table 1 shows the output structures and activation sizes of all layers.

The kernel size (3 × 3) is in fact in the form of (h, w, d) *i.e.,* (3 × 3 × 1). The number of the trainable parameters at each layer is the dot product of h, w, and d. Additionally, a bias is added to each feature to adjust the output alongside the weighted sum of the inputs. The number of trainable parameters at a convolution layer is calculated as ((h * w * d) +1) * k), where k is output features and d is the dimension of the previous layer. A general formula for calculating the number of trainable parameters in each layer using Eq. (2).

(2) \begin{eqnarray*}{Z}_{n}=\sum _{i=0}^{j-1}({f}_{i}\ast {x}_{n-i})+b\end{eqnarray*}

where, *f* is the current kernel size, *x* is the previous layer output, *b* is a bias constant, and *z* is the output vector.

Currently, the trainable parameters in the first convolution layer *i.e.,* ((3 * 3 * 1) +1) * 32 = 320. Table 2 shows the number of trainable parameters in each layer of the CNN model.

A max-pooling operation was performed after the convolution layer for reducing the dimensionality of the image. A max-pooling is used to capture the most important features (Gambäck & Sikdar, 2017) from the feature maps. In this layer, the maximum number of predominant features are selected from the previous feature map hold by a filter and output the most prevalent features (GeeksforGeeks, 2022). In this model, the max-pooling of slide size (2 × 2) was used for dimensionality reduction which formed a layer of size 16 × 16 × 32 as shown in Table 1.

**Table 1:** The output structure and size of layers.

No.	Layer	Output shape	Size
1.	Input layer	32, 32, 1	1,024
2.	Convolution 1	32, 32, 32	32,768
3.	Max-pooling1	16, 16, 32	8,192
4	Batch normalization	16, 16, 32	8,192
4.	Convolution 2	16, 16, 64	16,384
5.	Max-pooling 2	8, 8, 64	4,096
6.	Batch normalization	8, 8, 64	4,096
7.	Convolution 3	8, 8, 128	8,192
8.	Max-pooling 3	4, 4, 128	2,048
9.	Batch normalization	4, 4, 128	2,048
10.	Fully connected layer 1	32	—
11.	Fully connected layer 1	28	—

**Table 2:** CNN layers and trainable parameters.

No.	Layer	Trainable parameters
1.	Input layer	0
2.	Convolution 1	320
3.	Max-pooling1	0
4	Batch normalization	128
4.	Convolution 2	18,496
5.	Max-pooling 2	0
6.	Batch normalization	256
7.	Convolution 3	3,856
8.	Max-pooling 3	0
9.	Batch normalization	512
10.	Fully connected layer 1	65,568
11.	Batch normalization	128
12.	Fully connected layer 1	924
Total trainable parameters		159,676

Moreover, a batch normalization (shown in Eq. (3)) operation was also performed after pooling to normalize the results of the preceding layer which permits independent learning of each layer in the network. According to Santurkar et al. (2018) the mechanism of batch normalization aims to make steady the inputs distribution to the network layer in a training time. Similarly, the dropout regularization layer (Poernomo & Kang, 2018) was also added for handling the overfitting problem.

(3) \begin{eqnarray*}{x}^{N}= \frac{x-mean(x)}{std(x)} .\end{eqnarray*}

Similarly, 64 features, kernel size (3 × 3), same-padding, and *s* = 1, were used in the second convolution layer which resulted an output layer of shape 16 × 16 × 64 and activation size of 16,384 elements as shown in Table 1. The number of parameters of this layer calculated using Eq. (2) yields a total of 18,496 trainable parameters as shown in Table 2. Again, the activation function used was ReLu as shown in Eq. (1) for handling the linearity problem. After the second convolution, the max-pooling of size (2 × 2) was used for dimensionality reduction. Batch normalization and dropout layers were added the same as in the first layer.

In the third convolution, 128 features, kernel size (3 × 3), same-padding of size 1, *s* = 1, and ReLu activation function as in Eq. (1) were used. The output shape of the third layer was of shape 8 × 8 × 128 and activation size of 8192 elements as shown in Table 1. The total number of trainable parameters as per Eq. (2) is 3856 as shown in Table 2. A max-pooling was used for dimensionality reduction and batch normalization was utilized for normalizing the results of the previous layer. At the end, a dropout regularization was used to handle the overfitting problems.

Within the CNN model, after analyzing the convolution layer, the flattening layer is used to vectorize the feature map and pass it to the connected layers. The output of the flatten layer is used as an input for the connected layers. Normally, there is no such special operation performed, but the only thing to do in this layer is to vectorize the feature map. Thus, the flatten vector size achieved was 2048.

The final step in the CNN model is the fully connected layers. This is worth mentioning that fully connected layers are consisted of several layers depends on the problem solution in which each neuron of one layer is connected to the neurons of the subsequent layer (Altwaijry & Al-Turaiki, 2021). In this model, the fully connected layer was built of size 32 neurons. The activation function used in the fully connected layer was ReLu as in Eq. (1) except in the output layer where soft-max was used instead as shown in Eq. (4). The fully connected layer was followed by batch normalization and dropout layers. The output layer or soft-max layer consisted of 28 neurons used to classify each letter based on the predicted value.

(4) \begin{eqnarray*}\sigma \left( {Z}_{i} \right) = \frac{{e}^{{z}_{i}}}{\sum _{j=1}^{N}{e}^{{z}_{j}}} .\end{eqnarray*}

Moreover, for the optimization of the proposed model, this study has tested three different optimizers: RMSProp, Adam, and Adagrad. The test results in Adam were more promising; therefore, the proposed model was optimized using the Adam optimizer. The categorical cross-entropy is used for measuring loss in predicting multi-class labels.

Subsequently, the model was trained using the Adam optimizer. The training set was divided into several batches, where each batch size consisted of equal number of samples. Moreover, the model was tested using different number epochs. The final optimal epochs were set to 50 and run the experiment on a Google Colab notebook with a GPU environment. The performance of the proposed model was evaluated using various evaluation measures.

### Experiment

**Figure 6:**
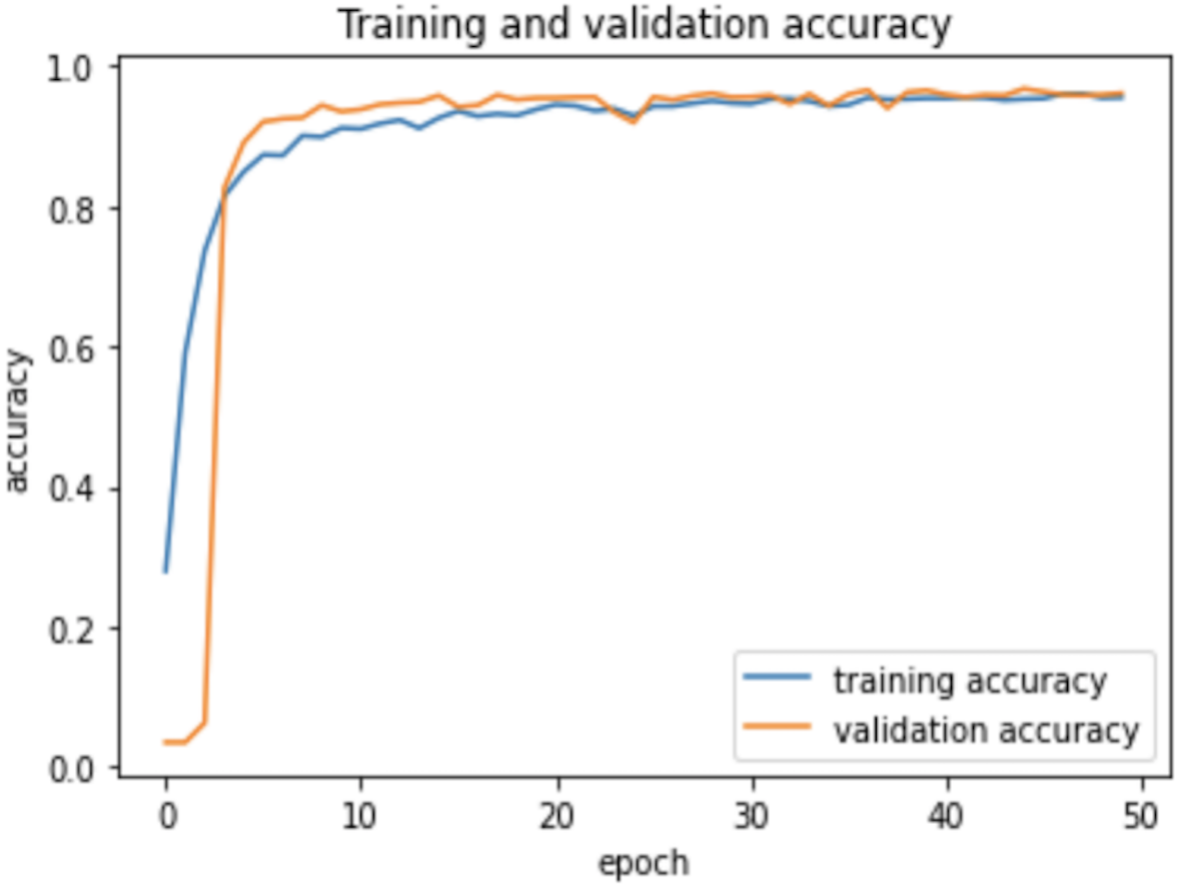
Learning accuracies with respect to epochs.

**Figure 7:**
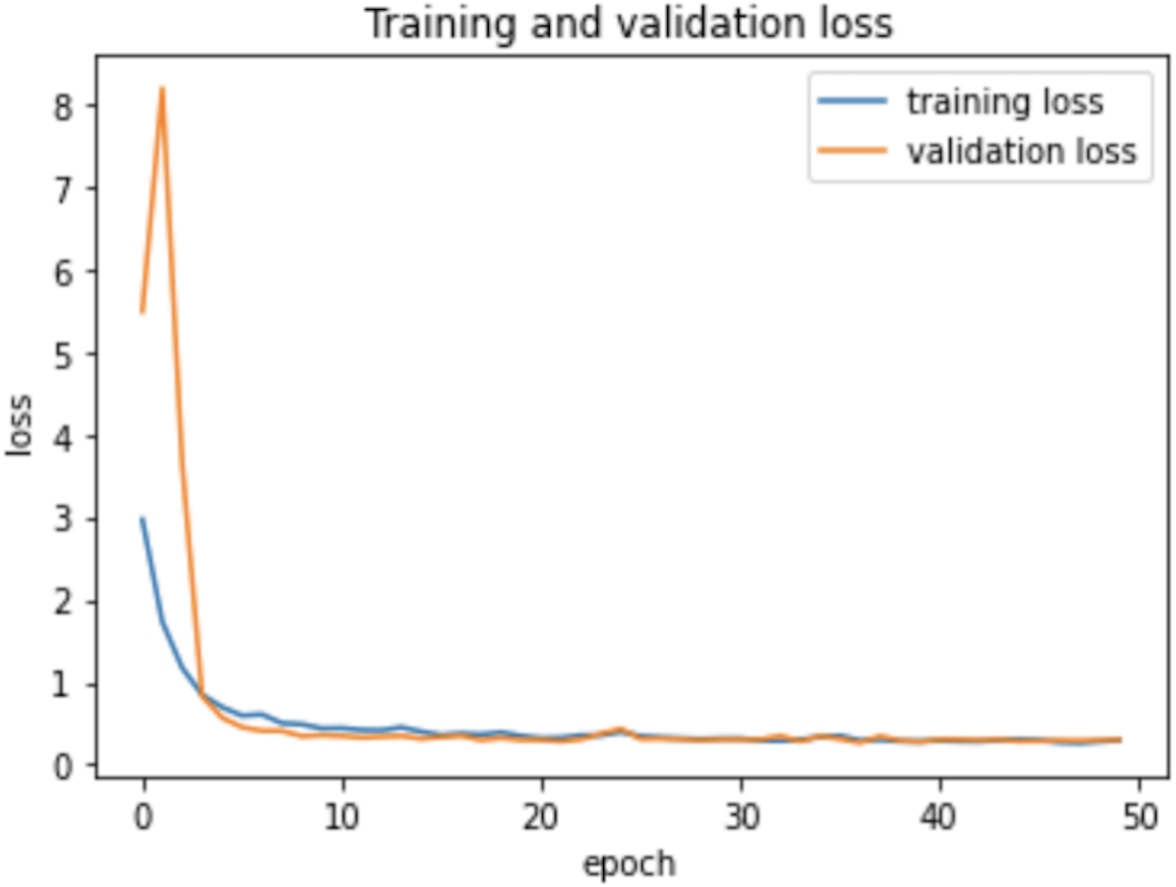
Learning losses with respect to epochs.

As the AHCD dataset is consisted of 16,800 images of which 80% of images contained in the training set, while 20% remained in the testing set. In the experiment of this study, the training set was further divided into two sets: 70% of the images for training and 30% for validation. The model was created using Adam optimizer, the loss was measured using categorical cross-entropy, and the metric was set to accuracy. The model was trained using 2 GHz Quad-Core Intel Core i5 on a Google Colab notebook with a GPU environment. The summarized model shows that the total trainable parameters were 159,676 as shown in Table 2. There were several attempts made with different numbers of epochs to achieve the best fit model; however, the epochs number set to 50 resulted in an optimal model for Arabic handwritten letter recognition. The average time of each epoch during training the model was around 9 s. Figure 6 shows the training and validation accuracies with respect to epochs. Normally, the training accuracy is high but both curves in Fig. 6 are very near to each other. Figure 7 shows the losses of training and validation. From Fig. 7, it has shown the lack of overfitting because as both curves advance, they coincide. Moreover, the overfitting problem was handled using dropout regularization which is supported by several studies (Baldi & Sadowski, 2013; Xu & Liu, 2020; Liang et al., 2021). During training, each node in the training epoch shows a probability parameter that is set by the dropout to be preserved and will be set to 0 otherwise (Xu & Liu, 2020).

**Figure 8:**
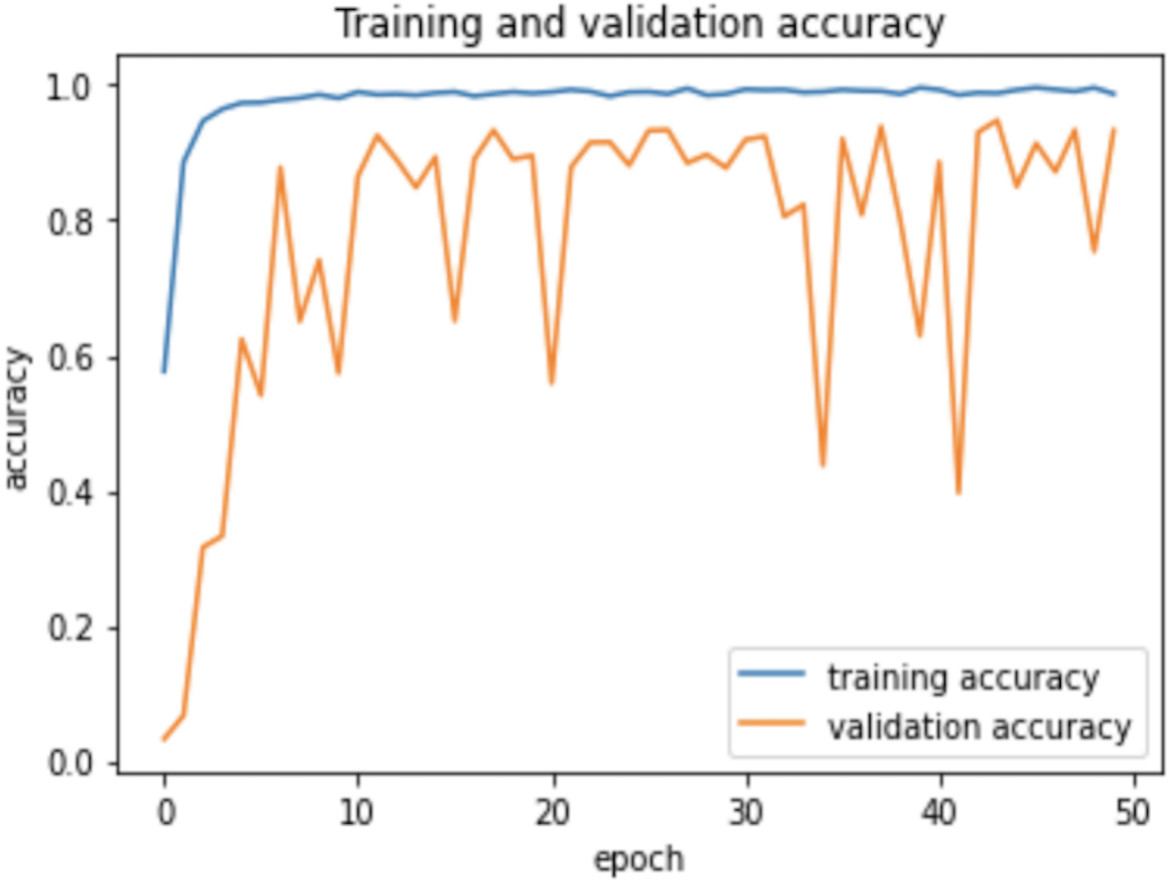
Learning accuracies without dropout.

**Figure 9:**
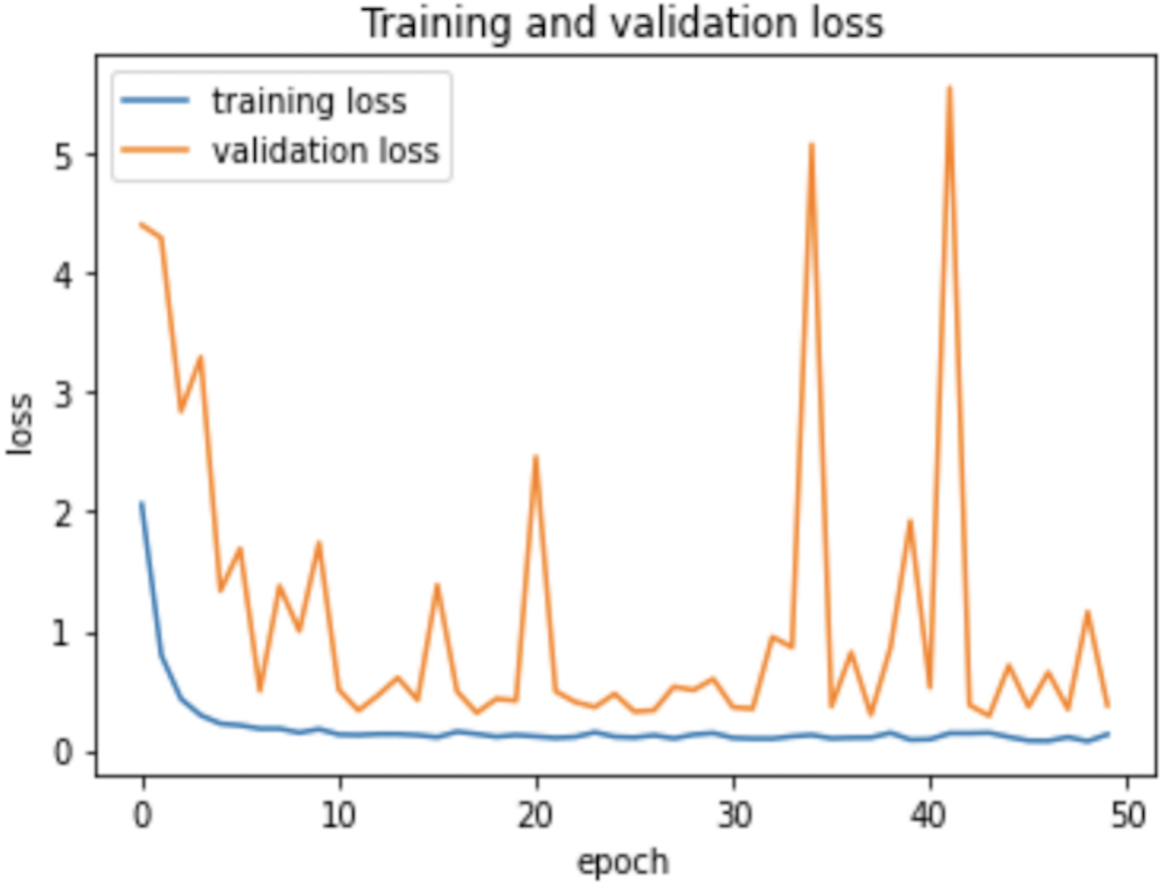
Learning losses without dropout.

Therefore, the dropout operation is widely used for preserving the probability parameter of each node in the training epoch, resulting in handling the overfitting problems. Figs. 8 and 9, show the accuracies and losses of training and validation curves respectively when the dropout was not used. In Fig. 8, the validation accuracies have deviated from the training curve. Similarly, Fig. 9 shows the divergence of the validation curve towards up starting from the first epoch, showing the model is overfitted. Comparing Figs. 9 to 7 above, the proposed model built with dropout regularization is best fitted.

## Results and Discussion

The performance evaluation of the proposed model was measured using accuracy, precision, recall, and f-measure using the following equations.

− The proportion of correctly recognized images to the entire number that is predicted (Ullah et al., 2021). Equation (5) shows computes accuracy.

(5) \begin{eqnarray*}Accuracy= \frac{TP+TN}{TP+TN+FP+FN} \end{eqnarray*}

− Precision is the proportion of relevant images among the retrieved images (Ali et al., 2021) which is computed using Eq. (6).

(6) \begin{eqnarray*}Precision= \frac{TP}{TP+FP} \end{eqnarray*}

− A recall is the proportion of relevant images that were retrieved (Ullah et al., 2021) which is computed using Eq. (7).

(7) \begin{eqnarray*}Recall~or~Sensitivity= \frac{TP}{TP+FN} \end{eqnarray*}

− The weighted average of precision and recall is known as the f-measure (Ullah et al., 2021) which is computed using Eq. (8).

(8) \begin{eqnarray*}\mathrm{F}-measure= \frac{(2\ast Precision\ast Recall)}{Precision+Recall} .\end{eqnarray*}

The overall accuracy of the proposed model for Arabic letter recognition is 96.78%. The model was also evaluated using other measures, such as precision, recall, and F-measures as shown in Table 3. The precision or confidence of a model is a ratio of correctly classified positive values to the total positive classified values (Ullah et al., 2021). The precision or positive predictive value (PPV) is calculated using Eq. (6). Recall or sensitivity of a model is calculated using Eq. (7), which is the ratio of correctly classified positive values to the whole values in the actual class (Ahmad et al., 2021). Similarly, F-measure as per Eq. (8) is the weighted average of PPV and sensitivity (Ullah et al., 2021; Al-Mudimigh, Ullah & Alsubaie, 2011).

**Table 3:** Model classification report.

Index No.	Class (Arabic letters).	Precision.	Sensitivity.	F-measure
0	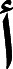	0.98	1.00	0.99
1	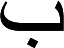	0.98	0.99	0.99
2	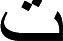	0.97	0.92	0.94
3	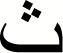	0.94	0.96	0.95
4	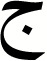	0.98	0.99	0.99
5	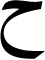	0.97	0.95	0.96
6	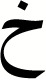	0.95	0.97	0.96
7	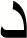	0.96	0.97	0.97
8	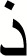	0.92	0.95	0.93
9	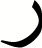	0.94	0.99	0.97
10	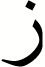	0.97	0.89	0.93
11	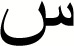	0.99	0.98	0.99
12	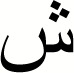	0.98	1.00	0.99
13	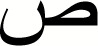	0.96	0.99	0.98
14	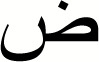	1.00	0.93	0.97
15	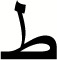	0.96	0.99	0.98
16	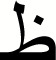	0.99	0.96	0.97
17	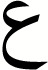	0.98	0.97	0.98
18	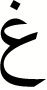	0.97	0.97	0.97
19	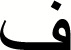	0.94	0.97	0.95
20	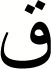	0.93	0.95	0.94
21	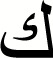	0.97	0.97	0.97
22	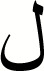	1.00	1.00	1.00
23	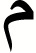	1.00	0.97	0.99
24	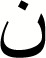	0.93	0.95	0.94
25	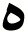	0.97	0.97	0.97
26	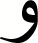	0.96	0.96	0.96
27	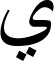	0.99	0.98	0.99
Accuracy				0.97
Macro avg		0.97	0.97	0.97
Weighted avg		0.97	0.97	0.97

The classification report in Table 3 shows promising results of the proposed model in terms of PPV, sensitivity and f-measures. In the proposed model, the average PPV, sensitivity, and f-measure are 96.71%, 96.75%, and 96.86% respectively. Moreover, individual letter values are shown in Table 3.

Comparing the result of our proposed model with the previous study exhibited in Table 4. El-Sawy, Loey & EL-Bakry (2017) have developed a CNN model containing two convolution layers of filter sizes 80 and 64 respectively. The convolution layers are followed by pooling layers. Regularization was used for handling overfitting and a fully connected layer of 1924 neurons was used followed by an output layer of 28 neurons. The model achieved a testing accuracy of 94.9% on the testing set. Our proposed intelligent model developed for recognizing the Arabic handwritten letters outperformed the El-Sawy, Loey & EL-Bakry (2017) model. The reported results showed an improvement rate of approximately 2% than the previous study. This enhancement in accuracy was due to using the different number of layers, regularization, number of iterations as well as the preprocessing steps discussed in the early sections. This is to be noted that the AHCD dataset was prepared by El-Sawy, Loey & EL-Bakry (2017) and also the first who tested AHCD using CNN. Therefore, we have compared our results with them.

**Table 4:** Comparison of the proposed model.

Study	Model	Dataset	Accuracy
El-Sawy, Loey & EL-Bakry (2017)	CNN	AHCD	94.9%
**Proposed Model**	CNN	AHCD	**96.78%**

**Figure 10:**
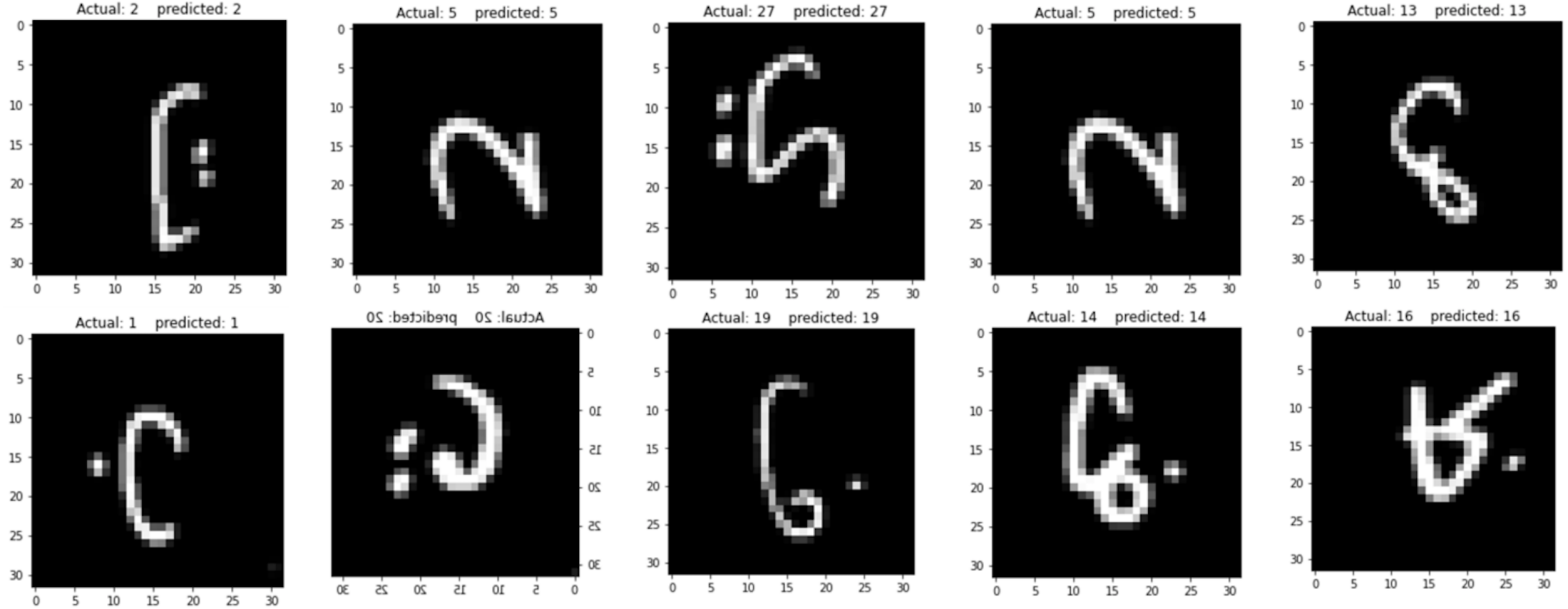
Model testing on unseen images.

Moreover, the proposed model has been tested on the unseen images and produced amazing results as shown in Fig. 10. In this step, the 10 images were randomly selected from a test set in the AHCD dataset. The proposed model printed the predicted and the actual indices for the 10 randomly selected images as shown in Table 5.

As in the data preprocessing stage, the indices of the images were reshaped that is starting from ‘0’ instead of ‘1’ in order for a better understanding of the classifier; therefore, in this study, the Arabic letters started from ‘0’. According to this standard indexing, the first letter which is ‘ 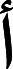
’ (alef) is at index ‘0’ and the last letter which is ‘ 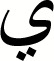
’ (yaa) at index ‘27’. As per Table 5, indices 2, 5, 27, 13, 5, 1, 20, 19, 14, and 16 represent ‘ 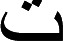
’ (taa), ‘ 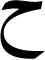
’ (haa), ‘ 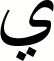
’ (yaa), ‘ 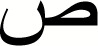
’ (sad), ‘ 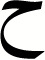
’ (haa), ‘ 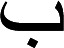
’ (baa), ‘ 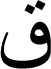
’(qaf), ‘ 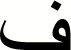
’ (faa), ‘ 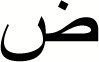
’ (dad), and ‘ 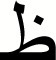
’ (dhaa) respectively. The corresponding test results of the proposed model are shown in Fig. 10. As both the ground reality indices and the predicted indices are the same after testing the unseen images on the proposed model, reports the reliability of the proposed model for recognizing the Arabic handwritten letters.

**Figure 11:**
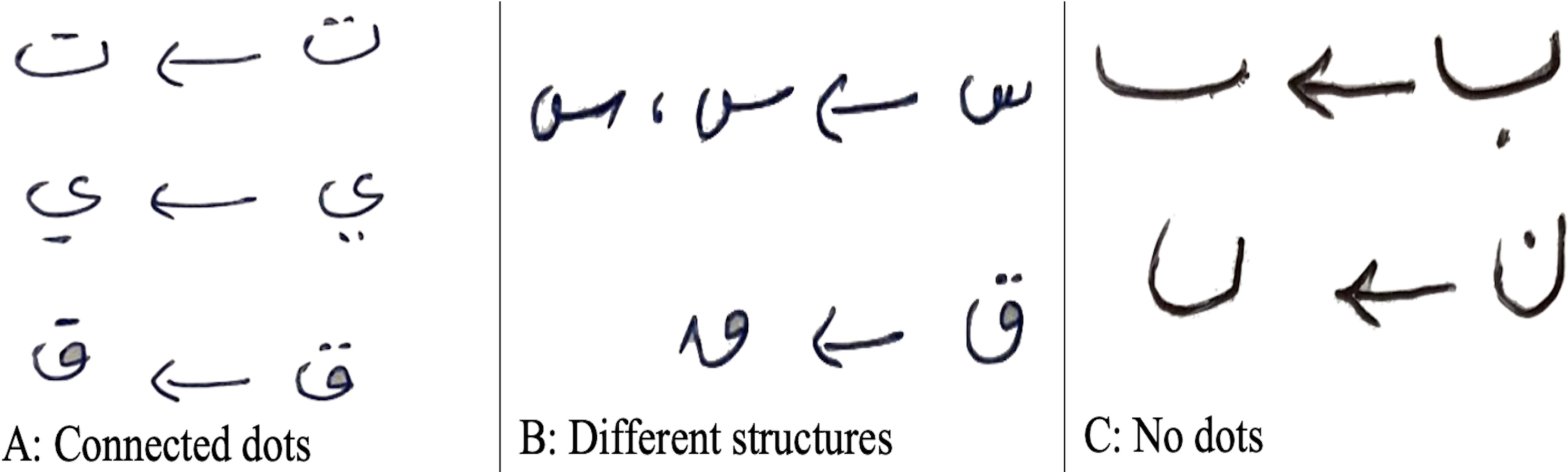
Letters representation in different forms.

The Arabic letter recognition is a challenge due to the similar structure of several letters such as a set of {‘ 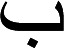
’ (baa), ‘ 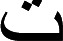
’ (taa), ‘ 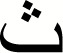
’ (thaa)} which can only be differentiated with the dot(s) at the top or bottom of the letter. The positions of these dots are not necessarily to be in the same position with every person because sometimes these dots are written very confusing like three dots in ‘ 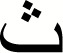
’ are closely similar to the letter ‘ 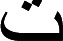
’ or the dot of letter ‘ 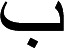
’ comes very closer to the letter itself and form a new shape or sometimes the two dot letter ‘ 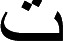
’ is written very light, showing just one dot, ultimately resembling the letter ‘ 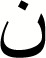
’(noon). It is worth mentioning that most Arabic native speakers write the two dots connected to each other and form a line (see Fig. 11A) which leads to an ambiguous situation, thus posing a challenge to digital recognition. Similar case for other sets like {‘ 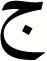
’ (geem), ‘ 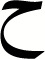
’ (haa), ‘ 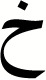
’ (khaa)}; {‘ 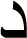
’ (dal), ‘ 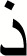
’ (thal)}, {‘ 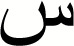
’ (sean), ‘ 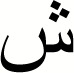
’ (shean)}, {‘ 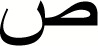
’ (sad), ‘ 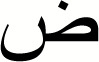
’ (dad)}, { ‘ 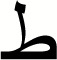
’ (taah), ‘ 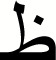
’ (dhaa)}, {‘ 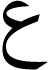
’ (aain), ‘ 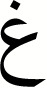
’ (ghain)}, etc. These challenges were also reported by El-Sawy, Loey & EL-Bakry (2017).

**Table 5:** Model testing on unseen images.

No. of images	10
Actual indices	2, 5, 27, 5, 13, 1, 20, 19, 14, 16
Predicted indices	2, 5, 27, 5, 13, 1, 20, 19, 14, 16

Another difficulty associated with Arabic letter recognition is the formation of a particular letter depending on an individual’s writing style. For example, the letters ‘ 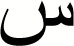
’ (sean) and ‘ 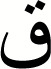
’(qaf) can be written in several forms, as shown in Fig. 11B. There are some letters as shown in Fig. 11C, which are by default understood to human; therefore, sometimes they write them without dots, but for the classifier, it is very difficult to differentiate and hence, lead to the confusion regarding not only the prediction of the original letters but between differentiating the letters ‘ 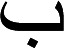
’ and ‘ 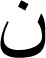
’ which are identical without dots (see Fig. 11C). Besides all these challenges, our proposed model is best fitted on AHCD dataset for recognizing the offline handwritten Arabic letters. Overall, the testing results are promising in terms of letter recognition.

## Conclusions and Future Work

This study proposed an intelligent approach based on CNN for recognizing handwritten Arabic letters. In this approach, regularization was utilized and the model overfitting was prevented using dropout operation, and batch normalization was used to normalize the outputs and allow independent learning of each layer in the network. The model was tested with and without dropout and found significant differences in the experimental results as shown in a set of figures (Figs. 6 and 7 *vs.* 8 and 9). In this study, ReLu was used as an activation function in all layers except the softmax layer and the model was created using the Adam optimizer. The experimental results shown in the above tables and figures reported the best fit of the proposed model. Moreover, the model was also tested on unseen samples and achieved excellent results (see Fig. 10). The comparison table shows the enhanced accuracy rate *i.e.,* 96.78% of the proposed model contributes to the reliability of the model best fit for the intelligent recognition of handwritten Arabic letters.

 As the handwritten scripts recognition is of active interest to several researchers. Therefore, in the future, the proposed model can be applied to other recognition tasks, such as the recognition of handwritten Arabic connected letters or script, Arabic digits, or handwritten alpha-numerical items occurring in similarly patterned languages, such as Urdu, Persian, Pashto, etc.

##  Supplemental Information

10.7717/peerj-cs.995/supp-1 Supplemental Information 1 Arabic Handwritten Characters

10.7717/peerj-cs.995/supp-2 Supplemental Information 2 Code

## Additional Information and Declarations

### Data Availability

The following information was supplied regarding data availability: The dataset used in this study is publicly available at Kaggle:
https://www.kaggle.com/mloey1/ahcd1.
